# The Degree of Plasma Oxidized Low-Density Lipoprotein Level Decrease Is Related to Clinical Outcomes for Patients with Acute Ischemic Stroke

**DOI:** 10.1155/2021/4998823

**Published:** 2021-12-14

**Authors:** Xiaoli Yang, Wenbo Sun, Duanlu Hou, Tianyao Wang, Chen Li, Yufan Luo, Shufan Zhang, Liwei Shen, Wenpeng Liu, Danhong Wu

**Affiliations:** ^1^Department of Neurology, Shanghai Fifth People's Hospital, Fudan University, Shanghai, China; ^2^Radiology Department, Shanghai Fifth People's Hospital, Fudan University, Shanghai, China

## Abstract

**Objective:**

To investigate the relationship between the decrease of plasma oxidized low-density lipoprotein (oxLDL) levels and clinical outcomes in patients with acute atherosclerosis-related ischemic stroke.

**Methods:**

We recruited acute ischemic stroke patients within 3 days of onset consecutively. Plasma oxLDL levels were measured on the second day after admission and before discharge (10-14 days after stroke onset). Initial stroke severity was assessed by the National Institutes of Health Stroke Scale (NIHSS) scores, and infarct volume was measured using diffusion-weighted imaging (DWI) by the ITK-SNAP software. Clinical outcomes were evaluated by DWI volumes in the acute phase, neurological improvement at discharge, and favorable functional prognosis at 90 days. Logistic regression was performed to evaluate the association between oxLDL level decrease and clinical outcomes.

**Results:**

207 patients were enrolled in this study. Compared with the mild decrease of the oxLDL level group, patients with a significant decrease of the oxLDL level group were more likely to have a higher ratio of neurological improvement at discharge (55.07% vs. 14.49%, *p* < 0.01) and favorable functional prognosis at 90 days (91.30% vs. 55.07%, *p* < 0.01). In multivariable logistic regression, the degree of oxLDL level decrease was related to neurological improvement at discharge and favorable functional prognosis at 90 days (*p* < 0.01). Patients with significant decrease were more likely to have neurological improvement at discharge (OR = 7.92, 95% CI, 3.14-19.98, and *p* < 0.01) and favorable functional prognosis at 90 days (OR = 7.46, 95% CI, 2.40-23.23, and *p* < 0.01) compared to patients with mild decrease of oxLDL level. The DWI volumes in patients with different oxLDL level decrease groups had no statistical difference (*p* = 0.41), and the Spearman's rho between oxLDL level decrease and DWI infarct volumes was -0.03, but no statistical difference (*p* = 0.72).

**Conclusions:**

The degree of oxLDL level decrease is related to neurological improvement at discharge and favorable functional prognosis at 90 days for patients with acute atherosclerosis-related ischemic stroke, but not with infarct volume in the acute phase.

## 1. Introduction

Atherosclerosis is the most common pathological mechanism of stroke [1–3]. Oxidized low-density lipoprotein (oxLDL) is the major product of lipid oxidative stress, with proinflammatory properties, that has been identified to mediate vascular endothelial cell dysfunction, activate platelet, and accelerate foam cell formation, resulting in the development of vulnerable atherosclerotic plaques eventually [2–7]. Therefore, oxLDL may be the trigger for atherosclerosis and contributes to clinical events onset as result [8–11].

Studies demonstrated that oxLDL in the acute phase was not only related to poor functional outcome after stroke [4, 12] but also can predict recurrent stroke in patients with minor stroke or transient ischemic attack independently, particularly atherosclerosis-related ischemic stroke, namely, in large artery atherosclerosis subtype and small-artery occlusion subtype [13]. Therefore, it is suggested that oxLDL may be a biomarker of stroke prognosis. However, the level of oxLDL is fluctuant in circulation. Study showed that it increased to the peak level on the third day and decreased gradually to the premorbid state [14]. To date, previous studies just focused on oxLDL at the acute stage of stroke [12, 13, 15], and only limited research has been conducted on the relationship between the decrease of oxLDL level and the prognosis of stroke, especially infarct volume. For this purpose, we analyzed acute infarct volumes using diffusion-weighted imaging (DWI) by the ITK-SNAP software and measured oxLDL level at different stages, to investigate the relationship between its level change with the infarct volume and the prognosis of stroke in patients with acute atherosclerosis-related ischemic stroke.

## 2. Methods

### 2.1. Study Population

This study was approved by the ethics committee of Shanghai Fifth People's Hospital. Written informed consent was obtained from all patients or their representatives. We prospectively recruited acute ischemic stroke patients within 3 days after onset who were admitted to our hospital between June 2018 and December 2020 consecutively. According to the purpose of this study, only large artery atherosclerotic and small-artery occlusion cerebral infarction (by following with the Trial of Org 10172 in Acute Stroke Treatment criteria [16]) were included in our analysis. Patients who met any one of the following criteria were excluded: (1) age < 35 years or >80 years; (2) history of stroke or coronary artery disease; (3) received thrombolysis or mechanical thrombectomy; (4) pregnant patients; (5) patients' severe heart failure, renal failure, liver failure, and respiratory failure; (6) patients with autoimmune diseases or tumor diseases; (7) the infection was associated with admission (such as urinary tract infection and pulmonary infection); and (8) no cerebral MRI.

### 2.2. Basic Clinical Data Collection

Demographic and clinical data were collected on admission by neurologists face-to-face, including age, sex, hypertension, diabetes mellitus, and other health conditions. Fasting venous blood samples to test for circulating ox-LDL levels, total cholesterol, low-density lipoprotein cholesterol (LDL-C), and other laboratory tests were obtained on the second day after admission and before discharge (10-14 days after stroke onset).

### 2.3. DWI Volumes

We followed the methods of Yang et al. [17]. All patients underwent multimodal MR imaging within 96 hours of admission. MR imaging examinations were performed using 3.0-tesla (Magnetom Skyra, Siemens, Germany). The imaging parameters were as follows: repetition time of 2800 milliseconds, echo time of 74 milliseconds, matrix number 220∗220 mm, voxel size 1.4∗1.4∗8 mm^3^, interslice gap 0.8 mm, and 2 *b* values of 0 and 1,000 s/mm^2^. Acute cerebral infarction lesions were defined as the presence of an increased signal on DWI, but with a corresponding low signal on the apparent diffusion coefficient (ADC) maps. The ITK-SNAP software (Version 3.8.0) was used to measure the acute infarct volumes indicated by DWI [18] (http://www.itksnap.org/pmwiki/pmwiki.php). The images were independently rated by 2 blinded stroke neurologists (Chen Li and Xiaoli Yang). 10 images were reviewed by them to assess interrater agreement, and interrater reliability was 0.88. The average infarct volume of two neurologists was used for analysis.

### 2.4. Prognosis of Stroke

Prognosis of stroke was evaluated by DWI volumes in the acute phase, neurological improvement at discharge, and favorable functional prognosis at 90 days. Neurological deficit was assessed by the National Institutes of Health Stroke Scale (NIHSS) scores [19]. According to the consensus of neurological practitioners, the degree of neurological deficit at the time of presentation was divided into three grades: mild, NIHSS score ≤ 4; moderate, 5 ≤ NIHSS < 10; and severe, NIHSS ≥ 10. Compared with the NIHSS score at the time of presentation, the clinical response at discharge was divided into 3 grades, NIHSS of 0 or ≥4 points of remission was defined as neurological improvement [20], and ≥2-point increase was defined as neurological worsening [21]. Modified Rankin scale (MRS) score was used to evaluate functional outcome at 90 days after stroke onset, where 0–2 were defined as the favorable outcomes and 3–6 were defined as poor outcomes. We followed up on all patients by telephone interview at 90 days after the initial stroke to assess the functional outcome.

### 2.5. Measurement of Circulating oxLDL

Fasting venous plasma samples were obtained on the second day after admission and before discharge (10-14 days after stroke onset) to measure the oxLDL level. The samples were centrifuged (2000 × g for 5 minutes) immediately after collection and were stored in a -80°C refrigerator. The oxLDL level was tested by a sandwich ELISA (Elabscience, China), which uses a monoclonal antibody specific for an epitope (monoclonal antibody 4E6) of the apolipoprotein B100 (ApoB 100) portion of the LDL particle by following the manufacturer's instructions (Elabscience, China). The intra-assay and interassay coefficient of variation of oxLDL level was 6.37% and 6.5%, respectively.

### 2.6. Statistical Analyses

The SPSS 21.0 software was used for statistical analyses. According to the change of oxLDL, the patients were divided into three groups on average. For each demographic and clinical feature, normal distribution continuous variables were presented as mean ± standard deviation, and abnormal distribution continuous variables were presented as median (interquartile range). The Student *t*-test or ANOVA for normally distributed parameters and Wilcoxon or Kruskal–Wallis test for nonparametric variables were used as appropriate. Categorical variables were expressed as frequency (percentage), and the *χ*^2^ test or Fisher exact test was used. Associations between oxLDL level degree and DWI infarct volumes were examined using Spearman rank correlation analysis. The association between oxLDL level degree and clinical outcome was performed by logistic regression. For all regression models, variables showing *p* < 0.2 on the respective univariate analyses were included in the models. A 2-sided *p* < 0.05 was considered significant.

## 3. Results

### 3.1. Baseline Characteristics of Study Patients

210 patients were included in this study, 3 patients were excluded because they were not available for follow-up, and consequently, 207 patients were included in the final analysis. According to the different degree of oxLDL level decrease, the patients were stratified into 3 groups on average: *Δ*tertile 1 (mild decrease, <33.33%), difference < 36.23 pg/mL; *Δ*tertile 2 (moderate decrease, 33.34%-66.66%), the difference was 36.23 pg/mL-60.63 pg/mL; *Δ*tertile 3 (significant decrease, ≥66.67%), the difference ≥ 60.64 pg/mL. There were no significant differences in age, hypertension, diabetes mellitus, LDL-C, and total cholesterol in patients with different oxLDL level decrease groups (*p* > 0.05). Patients with the mild decrease of the oxLDL level group were more likely to have a higher prevalence of tobacco use, but the *p* value was 0.05 (Table 1).

### 3.2. Clinical Course and Outcomes in Different oxLDL Level Decrease Groups

Comparisons of clinical course and outcome features between different oxLDL level decrease groups are presented in Table 2. Patients with a significant decrease of oxLDL level (*Δ*tertile 3) were more likely to have a lower ratio of severe neurological function impairment at admission (14.49% vs. 37.68%, *p* < 0.01) and higher neurologic improvement at discharge (55.07% vs. 14.49%, *p* < 0.01) compared with patients with mild decrease group (*Δ*tertile 1). Furthermore, with the decrease of oxLDL level, the proportion of good functional prognosis at 90 days gradually increased (55.07% vs. 84.06% vs. 91.30%, *p* < 0.01) (Table 2).

### 3.3. Association between Different oxLDL Decrease Groups and Clinical Outcomes

We investigated the association between different oxLDL level decrease groups and clinical outcomes. Decrease of oxLDL level was related to neurologic improvement at discharge and favorable functional prognosis at 90 days in multivariate regression models. Compared with patients with a mild decrease of oxLDL level, patients with a significant decrease were more likely to have a favorable functional prognosis at 90 days (OR = 7.46, 95% CI, 2.40-23.23, and *p* < 0.01) and neurological improvement at discharge (OR = 7.92, 95% CI, 3.14-19.98, and *p* < 0.01) after adjusting relevant confounding factors. The neurological impairment severity on admission was correlated with favorable functional prognosis at 90 days (*p* < 0.01), but not with neurological improvement at discharge (Table 3). The AUC of the model to predict favorable functional prognosis at 90 days was 0.89 (95% CI, 0.84-0.94, and *p* < 0.01) and to predict neurologic improvement at discharge was 0.78 (95% CI, 0.71-0.84, and *p* < 0.01).

### 3.4. The Relationship between the Decrease of oxLDL Level and the Infarct Volume

**Figure 1:**
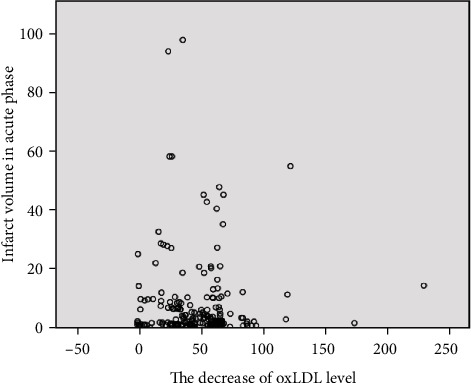
The scatter plot of the decrease of oxLDL level and DWI infarct volume in the acute phase.

The median DWI volumes in patients with the mild decrease group were 4.21 cm^3^, in the moderate change group was 3.08 cm^3^, and in the significant decrease group was 2.38 cm^3^, although presented reduced, with no statistical difference (Table 2). Therefore, the more significant the level of oxLDL decreases, but it does not mean that the infarct volume is smaller. Because the Spearman's rho between oxLDL level decrease and DWI infarct volumes was -0.03 (*p* = 0.72), there was no statistical difference; the scatter plot is shown in Figure 1.

## 4. Discussion

The major findings of our study were that the degree of oxLDL level decrease is related to neurological improvement at discharge and favorable functional prognosis at 90 days for patients with acute atherosclerosis-related ischemic stroke but was not correlated with infarct volume on DWI in the acute phase.

oxLDL is a biomarker of atherosclerosis [8, 11]. In the cardiovascular field, oxLDL has been designated as an independent risk factor for coronary artery disease [22] and was associated with increased mortality after acute myocardial infarction [23], so one study suggested oxLDL could be used in the diagnosis and evaluation of therapeutic effects in patients with coronary artery disease [24]. As the pathophysiological mechanisms of coronary and cerebrovascular atherosclerosis are considered similar, therefore, in the cerebrovascular field, recent studies have also shown that oxLDL level in the acute phase was not only related to poor functional outcome after stroke [4, 12] but also can predict recurrent stroke in patients with minor stroke or transient ischemic attack independently. However, the level of oxLDL is fluctuant in circulation. Study showed that it increased to the peak level on the third day and decreased gradually to the premorbid state [14]. To date, previous studies just focused on oxLDL at the acute stage of stroke [12, 13, 15]; only limited research has been conducted on the relationship between the change of oxLDL level with the prognosis of stroke. Our study extended previous studies and firstly reported that the significant change of oxLDL level is related to a favorable functional prognosis at 90 days for patients with acute atherosclerosis-related ischemic stroke.

Our study demonstrated that the degree of oxLDL level decrease is related to neurological improvement at discharge and favorable functional prognosis at 90 days, suggesting that the degree of plasma oxidized low-density lipoprotein level decrease is related to clinical outcomes for patients with acute ischemic stroke. However, the specific mechanism is not very clear, and potential reasons were as follows. Firstly, oxLDL as a result of oxidative modification of low-density lipoproteins has proinflammatory and proatherosclerotic properties by inducing endothelial dysfunction [25]. Endothelial dysfunction may contribute to the blood-brain barrier (BBB) impairment, leading to the permeability of the BBB; increased circulatory immune cells permeate and infiltrate the surrounding brain parenchyma. These immune cells secrete proinflammatory cytokines, deteriorating the injurious damage following stroke as result [26], which was confirmed by many studies, because they all showed a positive relationship between oxLDL levels and severe neurological deficits in patients with acute ischemic stroke [27, 28]. Secondly, endothelial dysfunction and continuous neuroinflammation state caused by oxLDL may accelerate atherosclerosis progression and promote the formation of unstable plaques, contributing to clinical events onset [9–11]. Studies suggested that there was a significant correlation between plasma and plaque oxLDL level [29], and high plasma levels of oxLDL were correlated with the vulnerability to rupture of atherosclerotic lesions [29, 30]. Therefore, oxLDL can be used as a biomarker of atherosclerotic plaque stability [31]. Given the volatility of oxLDL levels in the cycle [14], we only recruited acute ischemic stroke patients within 3 days after onset to ensure the initial level of oxLDL is at its peak level. In our study, compared with patients with mild changes of oxLDL level, patients with significant changes were more likely to have favorable functional prognosis at 90 days (OR = 7.46, 95% CI, 2.40-23.23, and *p* < 0.01) and neurological improvement at discharge (OR = 7.92, 95% CI, 3.14-19.98, and *p* < 0.01). Therefore, the more the level of oxLDL decreases, the more likely the plaque tends to be stable, and the more likely the neurological function will be improved.

We found that the change of oxLDL level is not related to infarct volume on DWI. The median DWI volumes in patients with different oxLDL change groups were 4.21 cm^3^, 3.08 cm^3^, and 2.38 cm^3^, respectively, although presented reduced gradually, with no statistical difference. Tsai et al. [32] demonstrated the oxLDL level in the acute phase was positively correlated with infarct volume. Uno et al. [28] evaluated oxLDL levels and ischemic lesions in acute stroke and confirmed that a persistent plasma oxLDL elevation was associated with enlargement of the ischemic lesion in the early phase after acute ischemic stroke. However, as far as we know, we are the first to explore the relationship between the change of oxLDL level and infarct volume on DWI.

Our study had some limitations. First, our sample size is relatively small, so given the biological characteristics of oxLDL, we only focus on acute ischemic stroke associated with atherosclerosis, namely, large artery atherosclerotic and small-artery occlusion cerebral infarction. Secondly, we recruited acute ischemic stroke patients within 3 days after onset; the initial oxLDL level was not the time of stroke onset. What is more, our oxLDL level for the second time was before discharge (10-14 days after stroke onset); it has not reached the level before the stroke onset [14], so the change of oxLDL level was between acute phase and subacute phase, but not the acute phase and premorbid state.

Despite all these, we evaluated the changes of plasma oxLDL levels and their impact on stroke prognosis over time and confirmed that the significant decrease of oxLDL levels is related to the neurological improvement at discharge and favorable functional prognosis at 90 days for patients with acute ischemic stroke associated with atherosclerosis [33]. These findings are more strongly confirmed that oxLDL was related to stroke prognosis and shed new light on oxLDL which may serve as a therapeutic target in improving functional outcomes after acute ischemic stroke simultaneously.

## Figures and Tables

**Table 1:** Demographic and baseline clinical characteristics of patients with different oxLDL degree groups.

	*Δ*tertile 1(mild decrease)	*Δ*tertile 2(moderate decrease)	*Δ*tertile 3(significant decrease)	*p* value
	Δ < 36.23 pg/mL	*Δ*: 36.23 pg/mL-60.63 pg/mL	Δ ≥ 60.64 pg/mL	
Age, y	65.04 ± 12.17	65.97 ± 10.88	68.74 ± 10.20	0.13
Male, *n* (%)	51 (73.91)	45 (65.22)	46 (66.67)	0.50
Alcohol use, *n* (%)	21 (30.43)	14 (20.29)	10 (14.49)	0.07
Tobacco use, *n* (%)	34 (49.28)	20 (28.99)	26 (37.68)	0.05
History of disease, *n* (%)				
Diabetes mellitus	21 (30.88)	31 (44.93)	24 (34.78)	0.21
Hypertension	52 (76.47)	55 (79.71)	50 (72.46)	0.61
TC, mmol/L	4.12 ± 1.09	4.30 ± 0.96	4.28 ± 1.07	0.55
TG, mmol/L	1.34 (0.84, 1.82)	1.40 (0.94, 1.88)	1.32 (1.02, 1.93)	0.59
HDL-C, mmol/L	1.01 (0.83, 1.23)	1.00 (0.84, 1.30)	1.00 (0.87, 1.24)	0.97
LDL-C, mmol/L	2.69 ± 1.04	2.74 ± 0.85	2.84 ± 0.91	0.63
CRP, mg/L	0.78 (0.19, 2.54)	1.14 (0.26, 3.91)	0.96 (0.39, 3.22)	0.62
Homocysteine, *μ*mol/L	14.45 (10.50, 19.10)	13.75 (11.70, 16.38)	15.10 (11.30, 18.60)	0.87

**Table 2:** Clinical course and outcomes in different oxLDL level decrease groups.

	*Δ*tertile 1(mild decrease)	*Δ*tertile 2 (moderate decrease)	*Δ*tertile 3(significant decrease)	*p* value
	Δ < 36.23 pg/mL	*Δ*: 36.23 pg/mL-60.63 pg/mL	Δ ≥ 60.64 pg/mL	
NIHSS score at admission (median)	6 (2.5, 8.5)	4 (2, 5)	3(2, 4)	<0.01
Neurological impairment severity at admission				<0.01
Mild, NIHSS score ≤ 4	23 (33.33)	28 (40.58)	40 (57.97)	
Moderate, NIHSS scores 5-9	20 (28.99)	35 (50.72)	19 (27.54)	
Severe, NIHSS score ≥ 10	26 (37.68)	6 (8.70)	10 (14.49)	
DWI volumes (cm^3^)	4.21 (1.17, 9.58)	3.08 (1.27, 4.73)	2.38 (1.59, 8.68)	0.41
Median onset-to-MRI time (hour)	74.20 (52.05, 99.00)	68.50 (45.75, 88.75)	66.00 (32.25, 97.00)	0.49
Clinical response at discharge				<0.01
Neurological improvement	10 (14.49)	29 (42.03)	38 (55.07)	
No significant neurological change	27 (39.13)	23 (33.33)	20 (28.99)	
Neurologic worsening	32 (46.38)	17 (24.64)	11 (15.94)	
Favorable functional prognosis	38 (55.07)	58 (84.06)	63 (91.30)	<0.01

**Table 3:** Logistic regression model for predictors of clinical outcomes.

	Neurological improvement at discharge				Favorable functional prognosis at 90 days			
	Unadjusted OR (95% CI)	*p* value	Adjusted OR (95% CI)	*p* value	Unadjusted OR (95% CI)	*p* value	Adjusted OR (95% CI)	*p* value
Age, y	0.98 (0.95, 1.00)	0.06	0.96 (0.93, 0.99)	0.02	0.99 (0.97, 1.02)	0.66	—	
Male, *n* (%)	0.53 (0.29, 0.96)	0.04	0.52 (0.23, 1.78)	0.12	0.87 (0.43, 1.77)	0.70	—	
Alcohol use, *n* (%)	0.71 (0.35, 1.44)	0.34	—		0.79 (0.37, 1.68)	0.53	—	
Tobacco use, *n* (%)	1.53 (0.85, 2.75)	0.16	1.52 (0.51, 2.60)	0.73	1.18 (0.61, 2.27)	0.62	—	
History of disease, *n* (%)								
Diabetes mellitus	1.26 (0.70, 2.25)	0.44	—		1.17 (0.59, 2.33)	0.65	—	
Hypertension	0.83 (0.43, 1.59)	0.57	—		0.71 (0.31, 1.58)	0.40	—	
TC, mmol/L	1.26 (0.95, 1.67)	0.10	0.83 (0.42, 1.63)	0.59	1.10 (0.80, 1.50)	0.58	—	
TG, mmol/L	1.08 (0.83, 1.41)	0.56	—		1.19 (0.83, 1.69)	0.35	—	
HDL-C, mmol/L	1.12 (0.46, 2.71)	0.80	—		0.63 (0.23, 1.68)	0.35	—	
LDL-C, mmol/L	1.42 (1.03, 1.94)	0.03	1.65 (0.77, 3.51)	0.2	1.19 (0.83, 1.71)	0.35	—	
CRP, mg/L	0.96 (0.86, 1.07)	0.47			1.15 (1.00, 1.33)	0.07	1.14 (0.95, 1.37)	0.15
Homocysteine, *μ*mol/L	0.96 (0.93, 1.02)	0.24	—		0.97 (0.94, 1.01)	0.12	0.97 (0.93, 1.00)	0.09
DWI volumes (cm^3^)	0.98 (0.95, 1.00)	0.08	0.98 (0.95, 1.01)	0.19	0.95 (0.92, 0.97)	<0.01	0.94 (0.91, 0.97)	<0.01
Neurological impairment severity at admission		0.06		0.45		<0.01		<0.01
Mild, NIHSS score ≤ 4	2.75 (1.18, 6.41)	0.02	1.80 (0.67, 4.81)	0.24	13.40 (5.32, 33.75)	<0.01	9.18 (3.27, 25.80)	<0.01
Moderate, NIHSS scores 5-9	2.36 (0.99, 5.65)	0.05	1.85 (0.68, 5.04)	0.23	6.30 (2.70, 14.71)	<0.01	6.43 (2.31, 17.85)	<0.01
Severe, NIHSS score ≥ 10	Reference		Reference		Reference		Reference	
Quartiles of oxLDL decrease		<0.01		<0.01				<0.01
*Δ*tertile 1 (mild decrease)	Reference		Reference		Reference		Reference	<0.01
*Δ*tertile 2 (moderate decrease)	4.28 (1.88, 9.74)	<0.01	3.64 (1.46, 9.08)	<0.01	4.30 (1.93, 9.58)	<0.01	2.63 (1.02, 6.83)	0.046
*Δ*tertile 3 (significant decrease)	7.23 (3.18, 16.44)	<0.01	7.92 (3.14, 19.98)	<0.01	8.57 (3.27, 22.43)	<0.01	7.46 (2.40, 23.23)	<0.01

## Data Availability

The datasets used and/or analyzed during the current study are available from the corresponding author on reasonable request.

## Abbreviations

oxLDL: Oxidized low-density lipoprotein
LDL-C: Low-density lipoprotein cholesterol
HDL-C: High-density lipoprotein cholesterol
DWI: Diffusion-weighted imaging
BBB: Blood-brain barrier
ADC: Apparent diffusion coefficient
NIHSS: National Institutes of Health Stroke Scale.
